# High-energy-density acoustofluidic device using a double-parabolic ultrasonic transducer

**DOI:** 10.1103/physrevapplied.23.024031

**Published:** 2025-02-12

**Authors:** Enrico Corato, Ola Jakobsson, Wei Qiu, Takeshi Morita, Per Augustsson

**Affiliations:** 1 Department of Biomedical Engineering, Lund University, Lund, Sweden; 2 Department of Precision Engineering, The University of Tokyo, Tokyo, Japan

## Abstract

High-acoustic-energy-density acoustofluidic devices are necessary to make this technology a viable option for clinical applications in the biomedical field. We present a mechanical interface that enables delivery of a high-amplitude acoustic field inside a fluid cavity by translating the vibrations from two large piezoelectric elements into a microfluidic chip. The study comprises both experimental characterization of a double-parabolic metallic acoustic waveguide and simulations of its working mechanism in two dimensions. We could focus 4.9-μm polystyrene particles at a flowrate of 5 ml/min, corresponding to an average retention time of 13.5 ms for particles in the actuated area. Moreover, we measured the acoustic energy density in the channel at stopped-flow condition, obtaining an average value of 1207 J*/*m^3^ and a maximum value of 2977 J*/*m^3^ with an input electrical power of 1.5 W. By comparing the simulation results with laser-Doppler vibrometer measurements, we confirmed that transverse sound waves play a significant role in the working mechanism of the double-parabolic structure, thus paving the way for further future optimization of the waveguide design.

## I Introduction

Microfluidic cell and particle manipulation by acoustic fields has been demonstrated for applications such as determination of hematocrit [1], cell washing [2,3], isolation of rare cells [4–7], separation of cell subpopulations [8–10], food analysis [11–13], among others [14]. Acoustofluidics offers a gentle [15–17], label-free, and precise way of handling suspended objects. The range of applications that can be addressed with this technology is closely linked with the ability to further increase the throughput and concurrently to manipulate objects in the submicron scale. Both these needs depend on the amplitude of the acoustic field, since higher acoustic energy density in the microresonator results in stronger forces on the suspended objects [18]. Achieving high vibration amplitudes by simply increasing the electrical power to the transducer prompts major issues, such as performance degradation in the piezoelectric material due to its nonlinear nature, and associated heat generation leading to unacceptably high temperatures in the microfluidic chip.

Optimizing the mechanical interface between the piezoelectric elements and the device can greatly increase device performance, as it could introduce stronger sound waves into the device and shape the vibration close to the ideal mode, resulting in higher acoustic energy. Furthermore, an interface can function as a thermal decoupler between the piezoelectric material and the fluid. Biological samples are notoriously sensitive to heat as they remain viable only in a narrow temperature region, and previous research has shown how the piezoelectric elements can act as heat sources, especially if driven at high voltages [19–21]. Moreover, temperature inhomogeneities in the fluid have been proven to drive thermoacoustic body forces in the presence of a sound standing wave [22,23], phenomenon that could potentially increase the critical particle size, below which particle migration is dominated by streaming, due to the induced fast thermoacoustic streaming [24].

Hard-type piezoelectric materials, such as hard lead zirconate titanate (PZT), are commonly used in bulk-acoustic-wave devices. To optimally drive an acoustofluidic system, one approach is to apply antisymmetric actuation to a piezoelectric element mounted to the device bottom [25], resulting in enhanced vibration of the channel side walls. Recently, Qiu *et al*. have demonstrated that the placement of a piezoelectric element is crucial for obtaining high acoustic energy in the channel [26]. By confronting bottom and side actuation for standard bulk-acoustic-wave devices with sound fields in the channel-width direction, they found that the latter is at least 4 times more efficient. Regarding mechanical interfaces, previous works have employed wedges to couple the piezoelectric element with the acoustofluidic chip [27]. By changing the angle of the incidence wave with respect to the chip, it was later reported that it is possible to always hit some chamber resonance, but with the acoustic energy dependent on the wedge angle [28]. However, the reported acoustic-energy-density values were rather low, and the system required frequency modulation to generate a nodal line in the channel center.

In the power ultrasonics field, such as ultrasonic cleaning [29], welding [30], and cutting [31], Langevin transducers are widely used together with horn or radiator structures [32]. They are typically operated at the frequencies of a few tens of kHz and hence the dimensions of those transducers range between a few centimeters to a few tens of centimeters. Employing such transducers at MHz frequencies would require a reduction of their sizes down to a few millimeters, assuming the vibration mode to remain the same, which is not practical to integrate with acoustofluidic devices. Hence, a different approach must be taken to achieve high vibrations at high frequencies. A double parabolic wave-guided high-power ultrasonic transducer (DPLUS) [33] has been shown to deliver vibration velocities of its waveguide tip in the order of hundreds of mm/s in the low MHz range. This circular structure has two opposing different-sized parabolas with the same focal point with opposite concavity. One parabola has a focal length equal to 10 mm and the second one 0.5 mm. With this design, the larger parabola focuses longitudinal sound waves onto the smaller parabola, which guides them as plane waves onto a tip. Thanks to the considerable size difference between the two parabolas, the resulting vibration was magnified 10–40 times relative to the piezoelectric source [34]. While the circular geometry of the DPLUS makes it suitable for exciting strong acoustic fields in a single point, it is not compatible with acoustic flow-focusing devices that typically require the formation of a standing wave across the width of a long microfluidic channel.

**Fig. 1:**
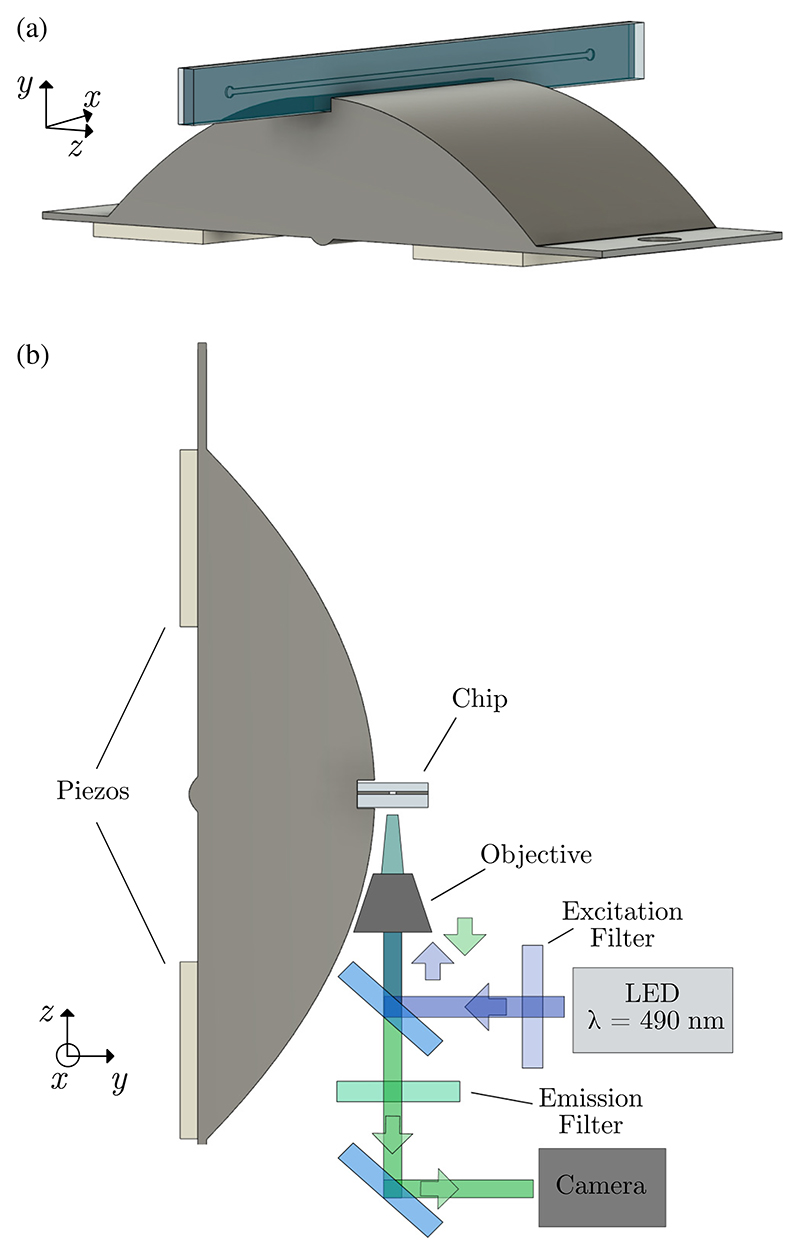
(a) Rendering of the device, showing the metallic LDPLUS structure, the piezoelectric elements glued at the bottom, and the chip glued on top. (b) Schematic of the device as mounted on the microscope.

In this work, we adapted the DPLUS design to excite a half-wavelength standing-wave field across the width of the channel in an acoustofluidic chip by extruding the parabolas along the length of the channel, Fig. 1(a). We investigated the operating principle by two-dimensional (2D) modeling of the device and we evaluated experimentally the device magnification coefficient by comparing the vibration in the slot where the chip sits to the vibration of the PZT elements. We evaluated the ability to focus 4.9-μm polystyrene particles at a high flowrate as a function of frequency and we measured the acoustic energy density in the channel along the actuation zone by particle tracking. With an average of 1207 J/m^3^ and a maximum of 2977 J/m^3^ achieved at an input power of 1.5 W, this is currently the highest acoustic energy density reported for acoustophoresis.

## II Materials and Methods

### A Device and system

The design is inspired by a circular double-parabolic device developed by Chen *et al*. [33]. Both devices have the same parabolic cross section, with the major difference being that our device has the cross section extruded for 20 mm along a line, instead of being axisymmetric, Fig. 1(a), and hence we refer to it as line DPLUS (LDPLUS). Furthermore, the structure has a slot on top to accommodate a microfluidic chip. The LDPLUS was fabricated in aluminum by computer-numerical-controlled milling (Datron Neo Series 2, Datron, Germany).

Two piezoelectric plates (Pz26, Ferroperm Piezoceramics, Meggitt A/S, Denmark) with dimensions of 20×10×1 mm^3^ and with a fundamental resonance frequency at approximately 2 MHz in their thickness mode were attached to the backside of the LDPLUS, extending along its whole length. The piezoelectric elements were glued with heat-curable epoxy (EpiFine, KISKO LTD, Japan) and we monitored their temperature by gluing a resistance temperature detector (Pt1000, JUMO GmbH & Co., Germany) on each element. The piezoelectric plates were driven with a function generator (AFG3022B, Tektronix, Inc., Beaverton, Oregon, USA), the signal of which was amplified by a power amplifier (75A250A, Amplifier Research, Pennsylvania, USA). Compressed air was blown to the back of the device for cooling.

The microfluidic chip (GeSim Bioinstruments and Microfluidics mbH, Germany) comprises a 150-μm-thick etched-through silicon layer, resulting in a 375-μm-wide and 40-mm-long channel. The silicon was anodically bonded to a glass top and bottom with thicknesses 500 and 760 μm, respectively. In the thicker layer, holes were drilled for an inlet and an outlet for the fluid. The channel was centered in the middle of the acoustofluidic chip, which is 4 mm wide and 50 mm long.

Liquid was infused into the channel by a syringe pump (neMESYS, Cetoni GmbH, Korbussen, Germany). A 10-ml glass syringe (Hamilton Company, Nevada, USA) ensured constant flowrate at the high pressure exerted by the pump. In the stopped-flow experiments, we employed a four-port, two-way manual diagonal valve (V-101D, IDEX Health & Science, Washington, USA) to short circuit the inlet and outlet and thus quickly stop the flow.

### B Acoustic focusing

The vibrations generate a first-order standing-wave field (*p*_1_) inside the fluid cavity, which in turn gives rise to time-averaged second-order effects, i.e., acoustic radiation forces (***F***_rad_) on particles [18,35] and acoustic streaming [36,37]. We assume a half-wavelength plane standing pressure wave *p*_1_ = *p*_*a*_ cos(*k*_*y*_ *y*)e^−i*ωt*^ with pressure amplitude *p*_*a*_, wave number *k*_*y*_ and angular frequency *ω*, across the channel width, with the pressure node located at the channel center (*y* = *W*/2). Considering a fluid with density *ρ*_0_ and compressibility *κ*_0_, and a particle of radius *a*, density *ρ*_*p*_, and compressibility *κ*_*p*_, the time-averaged acoustic radiation force acting on such a particle can be expressed as

(1a) $${\text{F}}_{\text{rad}}=4\pi a^{3}k_{y}E_{\text{ac}}\Phi (\tilde{\kappa },\tilde{\rho })\sin(2k_{y}y){\text{e}}_{y}$$

(1b) $$\Phi (\tilde{\kappa },\tilde{\rho })=\frac{1}{3}[\frac{5\tilde{\rho }-2}{2\tilde{\rho }+1}-\tilde{\kappa }],\tilde{\rho }=\frac{\rho_{p}}{\rho_{0}},\tilde{\kappa }=\frac{\kappa_{p}}{\kappa_{0}}.$$

Here *E*_ac_ = *p*_*a*_^2^*κ*_0_/4 is the time-averaged acoustic energy density, Φ is the so-called acoustic contrast factor, and **e**_*y*_ is the standard basis vector in the *y* direction.

For microparticles having Reynolds number well below unity, we can express the position-dependent particle velocity ***v***_*p*_ by balancing the acoustic radiation force ***F***_rad_ with the viscous Stokes drag force ***F***_drag_ = − 6*πηa****v***_*p*_, which results in

(2) $${\text{v}}_{p}=\frac{2a^{2}k_{y}E_{ac}\Phi (\tilde{\kappa },\tilde{\rho })}{3\eta }\sin(2k_{y}y){\text{e}}_{y}.$$

Here *η* is the dynamic viscosity of the fluid. In this work, we used polymer particles in water and an actuation frequency around 2 MHz, for which the contribution of acoustic streaming to the particle-focusing velocity is small and it can thus be neglected [36].

Using reference particles of known properties, from Eq. (2) we can estimate *E*_ac_ by fitting a sinusoidal function to the experimentally obtained particle-focusing velocities using *E*_ac_ and a phase shift as fitting parameters, as previously demonstrated [19,38].

### C Device characterization

The acoustic field in the acoustofluidic channel was characterized using an epifluorescence microscope (Eclipse Ti2, Nikon, Tokyo, Japan), equipped with a light-emitting diode having a center wavelength of 475 nm and a high-speed camera (Kinetix, Teledyne Photometrics, Arizona, USA) and a 4× objective (Nikon, Tokyo, Japan) with 0.13 numerical aperture and 16.5-mm working distance. An additional 1.5× intermediate magnifier lens was employed before the light-path changer of the microscope. Fluorescent polystyrene (PS) particles (G0500B, Fluoro-Max, Thermo Fisher Scientific, Waltham, Massachusetts, USA) with 4.9-μm nominal diameter were used to evaluate the acoustic field. To find the optimal operating frequency of the microchannel, we performed an in-flow experiment evaluating the focusing of 4.9-μm PS particles near the channel outlet, sweeping the frequency between 1.851 and 1.911 MHz at a constant flowrate of 5 ml/min and keeping an averaged peak-to-peak voltage of 30 V on both piezoelectric elements. The voltage and the current applied to each piezoelectric elements were measured via a Pico-Scope (5442D, Pico Technology, Cambridgeshire, United Kingdom). By computing the full width at half maximum (FWHM) of the intensity profile, we obtained a measure of the acoustic focusing for different frequencies. For each frequency, image acquisition started once the device reached a steady thermal state, namely a temperature variation of less than 0.2 °C on both piezoelectric elements, Appendix A, Fig. 8.

Once the optimal frequency had been identified, we quantified *E*_ac_ for that frequency, along the 20-mm-long region of the channel where the LDPLUS actuates the chip, by measuring the focusing velocity of 4.9-μm PS particles. We considered only the particles close to the midheight of the channel to avoid the effect of drag enhancement due to the presence of walls [39]. The particle motion was tracked under a stopped-flow condition at a frame rate of 4420 Hz using the MATLAB-based particle-tracking algorithm *DefocusTracker* [40–42]. The particles close to the focal plane were identified by cross-correlating their images to the training images containing the particles in optical focus. The particle tracking was performed in six contiguous sections along the channel length due to the limited field of view.

To characterize the LDPLUS, we measured the vibration velocity in the slot into which the microfluidic chip was to be glued, by laser-Doppler vibrometer (LDV) (VibroFlex, Politec GmbH, Germany) via a custom Lab-VIEW (National Instruments, Texas, USA) program. The characterization was conducted with the chip detached after the particle-focusing experiments. The device position was controlled via a three-axis motorized stage (MLJ250/M, Thorlabs GmbH, Germany; 6230V62000, PI, Massachusetts, USA; L505013212, PI, Massachusetts, USA). The piezoelectric elements were excited at the selected operating frequency for 11 different voltage amplitudes. The sinusoidal signal was generated by the function generator output of a PicoScope and amplified by the power amplifier. The applied voltage and current to the piezoelectric elements were measured by a voltage probe and a current probe, which were both recorded by the PicoScope together with the measured vibration velocity and displacement. The vibration was also measured at the backside of the two piezoelectric elements to relate their vibration to the slot vibration. Furthermore, we measured the transient vibration when actuating the piezoelectric elements with a burst wave with five cycles at the frequency and voltage leading to the best particle focusing. The transient excitation signal and the slot vibration velocity along the slot center line were both recorded by the PicoScope.

### D Numerical model

The LDPLUS was modeled in its 2D cross section using the commercial finite-element software COMSOL Multiphysics [43]. We used the software built-in materials, except for the piezoelectric material Pz26 and water, and their relevant material parameters are listed in Table I. First, we employed a frequency-domain study, using the *Solid Mechanics, Electrostatics*, and *Pressure Acoustics* modules, coupled via *Piezoelectricity* and *Solid-Acoustic interaction* multiphysics. We applied 1 V to the piezoelectric element as the harmonic load. The structure had a fixed-boundary condition on the two side flanges, while it was free to vibrate for all the other external boundaries. A convergence test with decreasing mesh element size assured a residual below 10^−3^, resulting in a maximum size of 13.5 μm in the PZT, 50 μm in the LDPLUS, 10 μm in the chip, and 2 μm in the channel. We assumed perfect coupling between the different domains, as no coupling layer was introduced, and aluminum, glass, and silicon are considered as linear elastic materials. Moreover, the piezoelectric material properties provided by the manufacturer are valid only under low input power and they are inevitably different at intermediate and high power due to nonlinear effect within the piezoelectric material itself. With these assumptions, we made qualitative comparisons between the simulation and the experiments. With the stationary solution we could identify the resonance frequency via a frequency sweep ranging from 1.8 to 2.0 MHz in 1-kHz steps. Second, we used a time-dependent solution with the *Elastic Waves-Time Explicit, Electrostatics*, and *Piezoelectricity* modules to visualize the wave path inside the structure without the chip on top. Thus, we actuated the piezoelectric elements with a burst wave of five cycles. We could also differentiate between longitudinal and transverse waves inside the structure by the sign of the second principal invariant of the stress tensor. The resulting vibration velocity on the slot top was compared with the experimental data measured via LDV with the same burst wave excitation.

## III Results

### A Experimental characterization

**Fig. 2:**
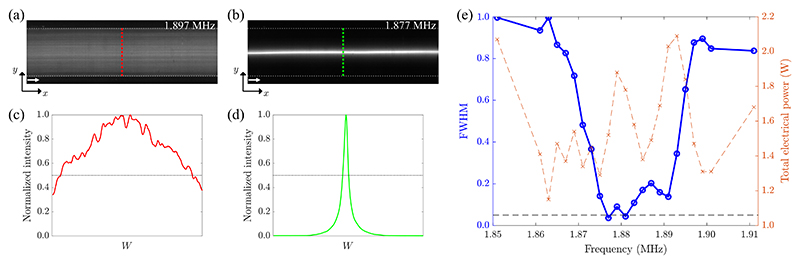
Flow-focusing characterization of the LDPLUS. (a),(b) Overlayed fluorescent images showing weak and strong focusing of 4.9-μm PS particle, respectively. The images were acquired close to the channel outlet and the arrows indicate the flow direction. The dashed gray lines show the channel walls. (c),(d) Fluorescence intensity profiles obtained by averaging images (a),(b) along the channel direction. The black dotted line indicates half of the maximum intensity. (e) FWHM of the fluorescence intensity profiles (blue circles) and total applied electrical power to both piezoelectric elements (orange crosses) measured in the frequency range from 1.851 to 1.911 MHz at a constant average voltage (30 V_pp_) over the piezoelectric elements. The black dashed line indicates 5% of the channel width.

In-flow focusing of 4.9-μm PS particles at a constant flowrate of 5 ml/min revealed the optimal operating frequency of the device. At this flowrate, the fluid has an averaged retention time in the actuated area of 13.5 ms. Figures 2(a) and 2(b) show the particle focusing near the channel outlet for weak and strong focusing, respectively. Videos 1 and 2 show the corresponding image sequence from which Figs. 2(a) and 2(b) were extracted. The corresponding intensity profiles are shown in Figs. 2(c) and 2(d), from which the FWHM was extracted. This value is measured in the frequency range from 1.851 to 1.911 MHz, Fig. 2(e), showing the focusing performance at each frequency after the temperature rise has converged. Furthermore, we measured the total electrical power consumed by both piezoelectric elements, so that we could discriminate the relative efficiency of the transducer when giving similar focusing. Hence, we selected 1.877 MHz as the optimal operating frequency, at which the FWHM of the band of focused particles was within 5% of the channel width with a total electrical power around 1.5 W.

**Fig. 3:**
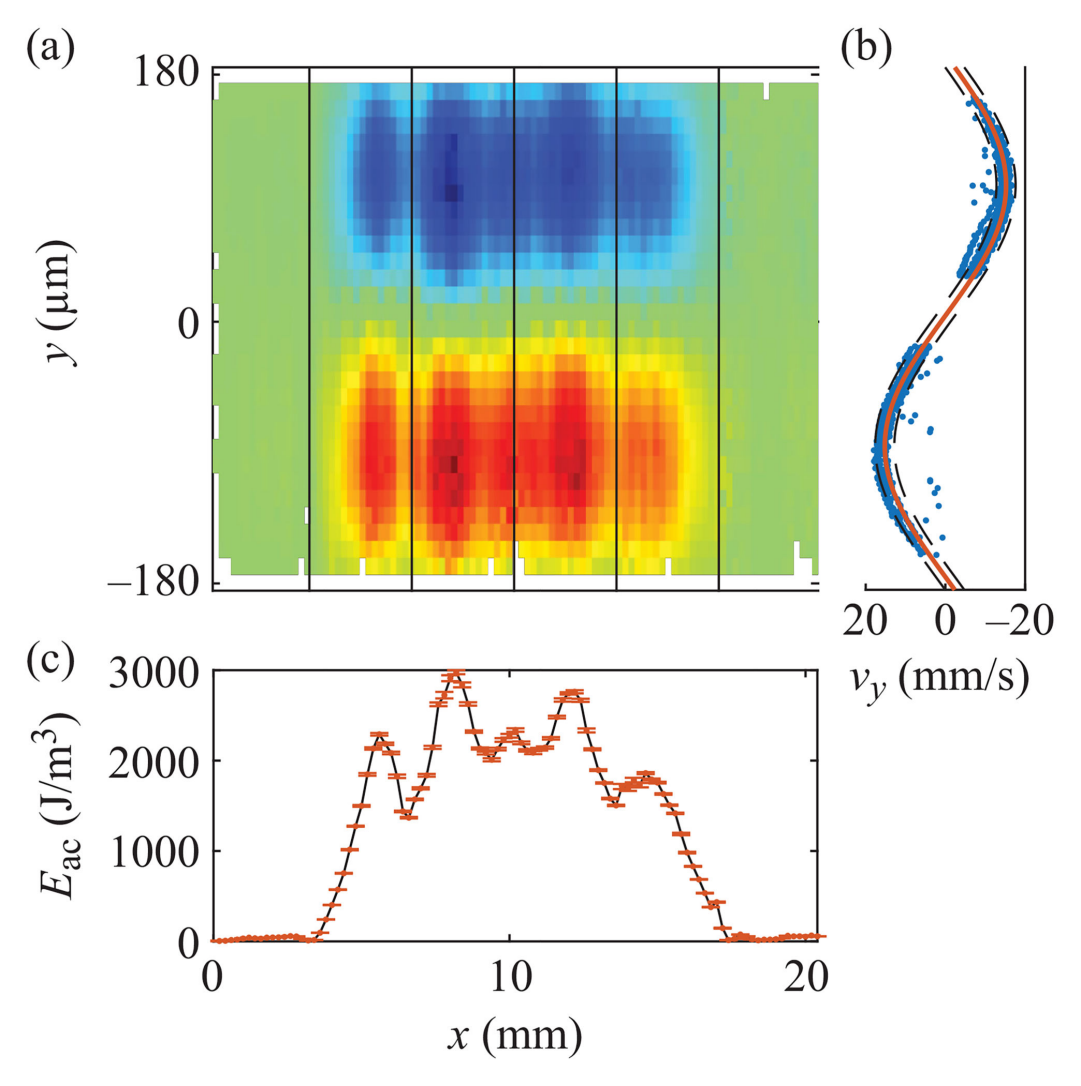
(a) Acoustic velocity field for 4.9-μm PS particles in the actuation zone of the channel (dark red corresponds to −30 mm/s, dark blue to 30 mm/s). (b) Example of a sinusoidal fitting (solid orange line) of the particle-focusing velocity along a 0.2-mm-long section of the channel. Dashed black lines indicate the 95% confidence interval. (c) Measured *E*_ac_ along the actuation zone. Error bars indicate the 95% confidence interval for *E*_ac_ (overlapping for most of the data points).

Having found that 1.877 MHz led to the best overall performance, we measured the acoustic energy density *E*_ac_ inside the channel at this frequency by tracking 4.9-μm PS particles at stopped-flow conditions. The particle-focusing velocity along the whole actuation zone in the channel is shown in Fig. 3(a). We observed focusing velocities on the order of several tens of mm/s, with a velocity field maximum value of 30.09 mm/s and an averaged value of 8.06 mm/s. By using Eq. (2), we fitted the velocity *y* component of the particles to a sinusoidal function every 0.2 mm along *x* direction, Fig. 3(b), and we thus obtained the spatial variation of *E*_ac_ along the whole actuation zone, Fig. 3(c). Clearly, the sound fields near the two ends of the transducer are very weak, while strong sound fields are excited in the center. We obtained a maximum *E*_ac_ of 2977 J/m^3^ and an averaged value of 1207 J/m^3^ along the whole actuation zone for a total input power of 1.5 W.

**Fig. 4:**
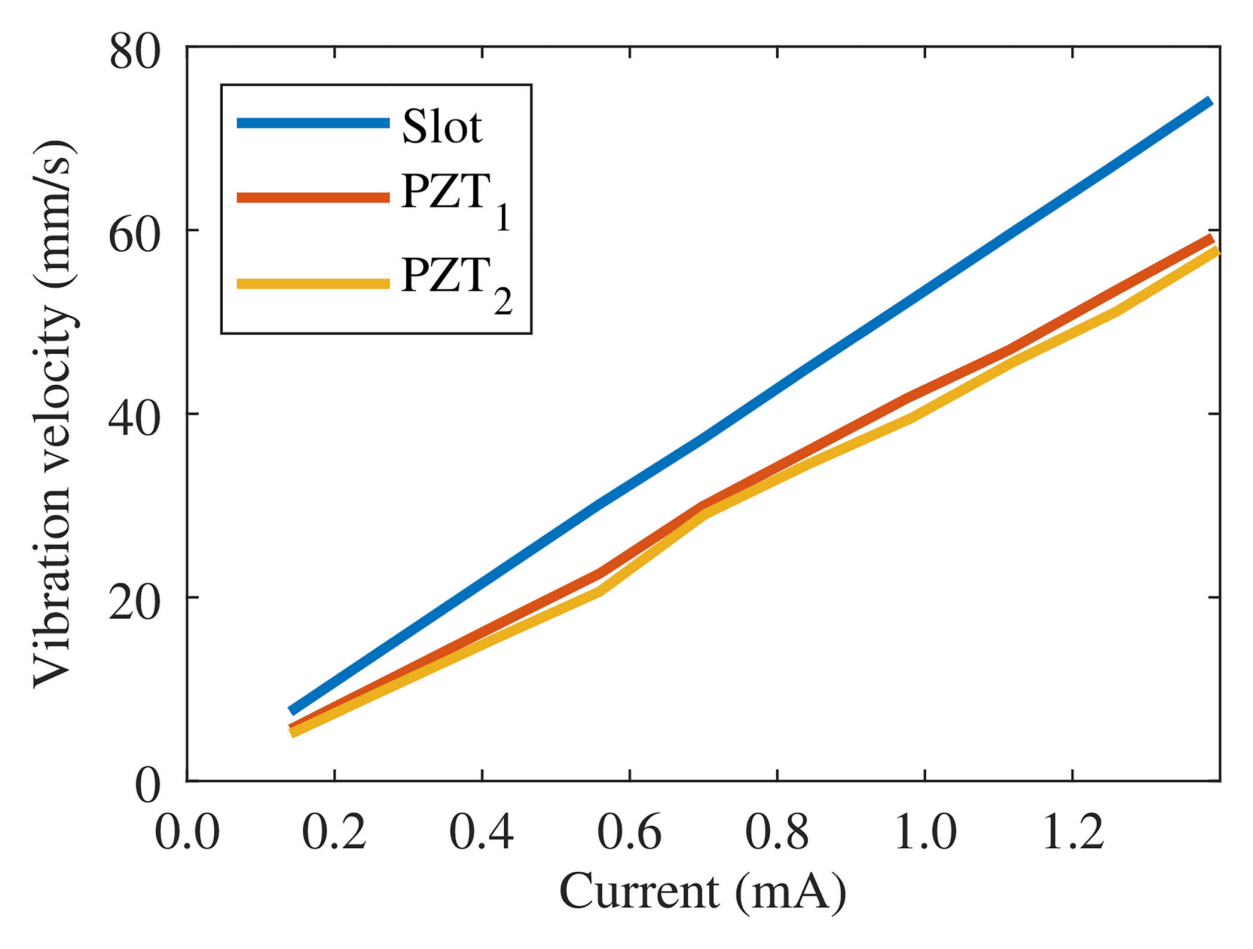
Mean vibration velocity in the LDPLUS slot (blue) and the two piezoelectric elements (orange and yellow) when actuated at 1.877 MHz. There was a linear relationship between the applied current to the piezoelectric elements and the mean vibration velocity. The slot always vibrated with a higher velocity amplitude than the piezoelectric elements, indicating that the LDPLUS was indeed able to amplify the vibration.

To quantify the magnification capability of our device, we measured the vibration of the back surfaces of the two piezoelectric elements, as well as that of the slot on top of the LDPLUS at 1.877 MHz. A linear relation between applied current to the piezoelectric elements and the mean vibration velocity of the surface is observed, as shown in Fig. 4. The slot was vibrating with a mean amplitude 1.25 times higher than that of the piezoelectric elements, indicating that the structure indeed translates vibrations to the chip that are comparable in magnitude to that of the piezo elements despite the higher complexity. The structure thus enables translation of sound while shielding the chip from excessive heating.

### B Numerical simulations

**Fig. 5:**
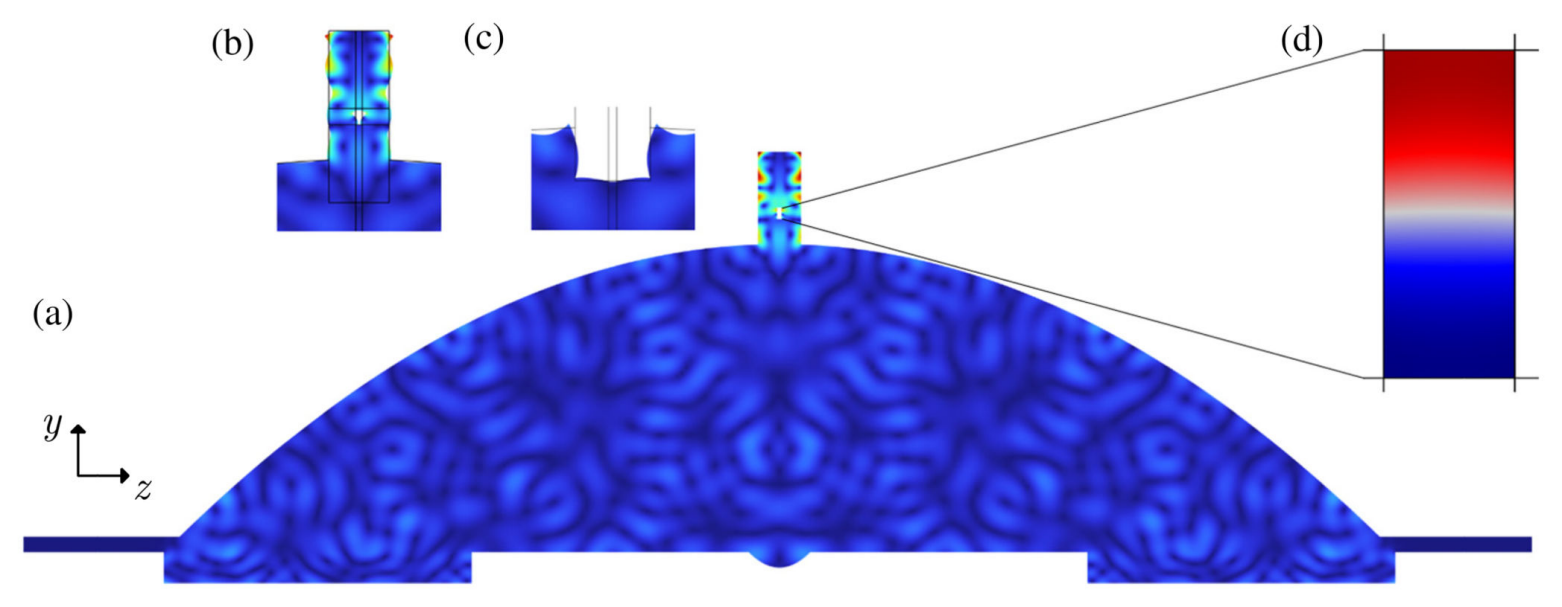
Frequency-domain simulation results at 1.860 MHz. (a) Vibration velocity in the solid ranging from zero (blue) to maximum (red). The highest absolute vibration velocities are in the microfluidic chip. (b) Displacement in the chip, with exaggerated deformation. (c) Displacement in the slot, with exaggerated deformation four times higher than in (b). (d) Enlargement of the water-filled microfluidic channel, showing a pressure standing wave with a single nodal line in the channel center (red-positive pressure, blue-negative pressure).

Numerical simulations were performed to understand the wave propagation and vibration mode in LDPLUS. We identified 1.860 MHz as the frequency resulting in the highest acoustic energy density inside the channel, whose steady-state vibration velocity is shown in Fig. 5(a). The highest vibration velocity in the entire structure occurs in the chip, instead of in the body of LDPLUS, which is desirable for inducing strong sound fields in the channel. This is further confirmed by plotting the displacement in the chip, shown in Fig. 5(b), which is much higher than in the slot, Fig. 5(c). The corresponding pressure field in the fluid chamber is shown in Fig. 5(d), where a pressure half-wavelength standing-wave field is observed with a nodal line in the channel center. We did not compare quantitatively this simulation to the experimental results, as it was performed with no coupling layers, linear materials, and low electrical power applied to the piezoelectric elements.

**Fig. 6:**
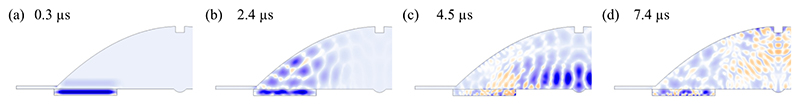
Transient behavior of the LDPLUS without the chip on top, with a five-cycle actuation at 1.877 MHz. The sign of the second principal stress invariant indicates longitudinal (blue) or transverse (orange) waves. (a) At 0.3 μs, longitudinal waves enter the LDPLUS structure. (b) At 2.4 μs, reflection on the big parabola. (c) At 4.5 μs, focused longitudinal waves are directed to the slot and the transverse waves lag behind the longitudinal waves. (d) At 7.4 μs, the structure vibration is clearly dominated by transverse waves.

To investigate the hypothesis of focusing and amplification of longitudinal waves by the double-parabolic structure, we evaluated the transient behavior of the LDPLUS with a time-domain simulation at 1.877 MHz, Fig. 6, which was the best actuation frequency as identified by the flow scan, Fig. 2. We chose this frequency for the simulation so as to compare with the experimental data leading to the best focusability. We investigated the wave propagation in the metallic structure for a burst excitation of five cycles by plotting the sign of the second principal invariant of stress, which enables a decomposition of longitudinal and transverse waves, resulting in positive values when the waves comprise mainly longitudinal components, negative when transverse waves dominate. Figure 6(a) shows how the piezoelectric material imposes longitudinal waves onto the structure at *t* = 0.3 μs. The first instances of mode conversion are visible in Fig. 6(b), as the longitudinal waves have been reflected by the big parabola, and the slot starts vibrating by the longitudinal waves coming radially from the piezoelectric elements. Figure 6(c) shows the longitudinal waves focused on the small parabola and directed to the slot, while the transverse waves are clearly lagging behind the longitudinal waves. At later times, the vibration in the LDPLUS becomes dominated by transverse waves, as shown in Fig. 6(d).

**Fig. 7:**
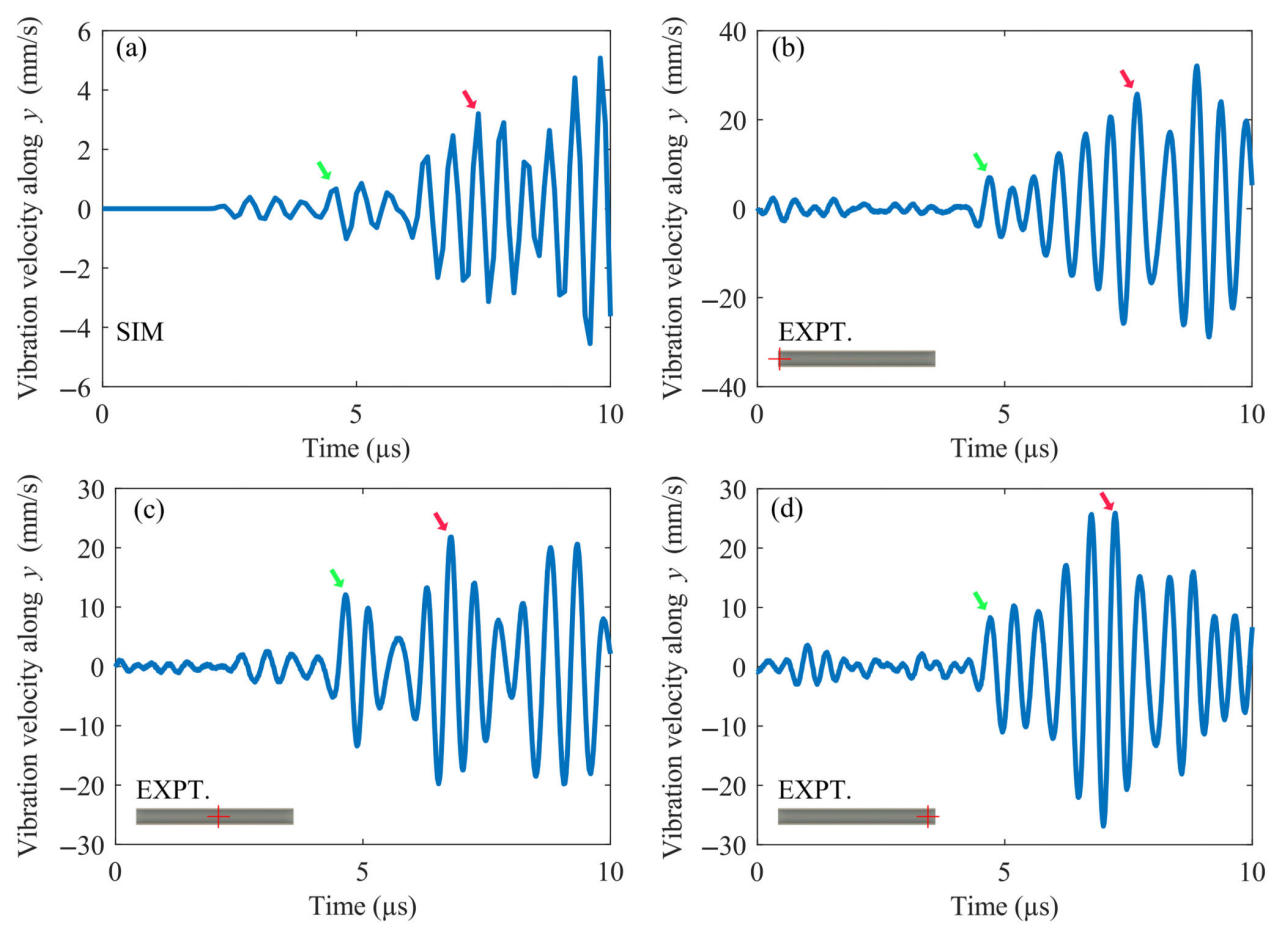
Comparison of the slot vibration velocity for the transient simulation (a) and the LDV measurements along the slot center line (b)–(d), displayed for the first 10 μs after the actuation onset. For experimental data, the red cross on the bottom left inset in each panel shows the measured position in the slot. The green arrows indicate the arrival of the focused longitudinal waves, while the red arrows point to the arrival of the transverse waves.

**Fig. 8:**
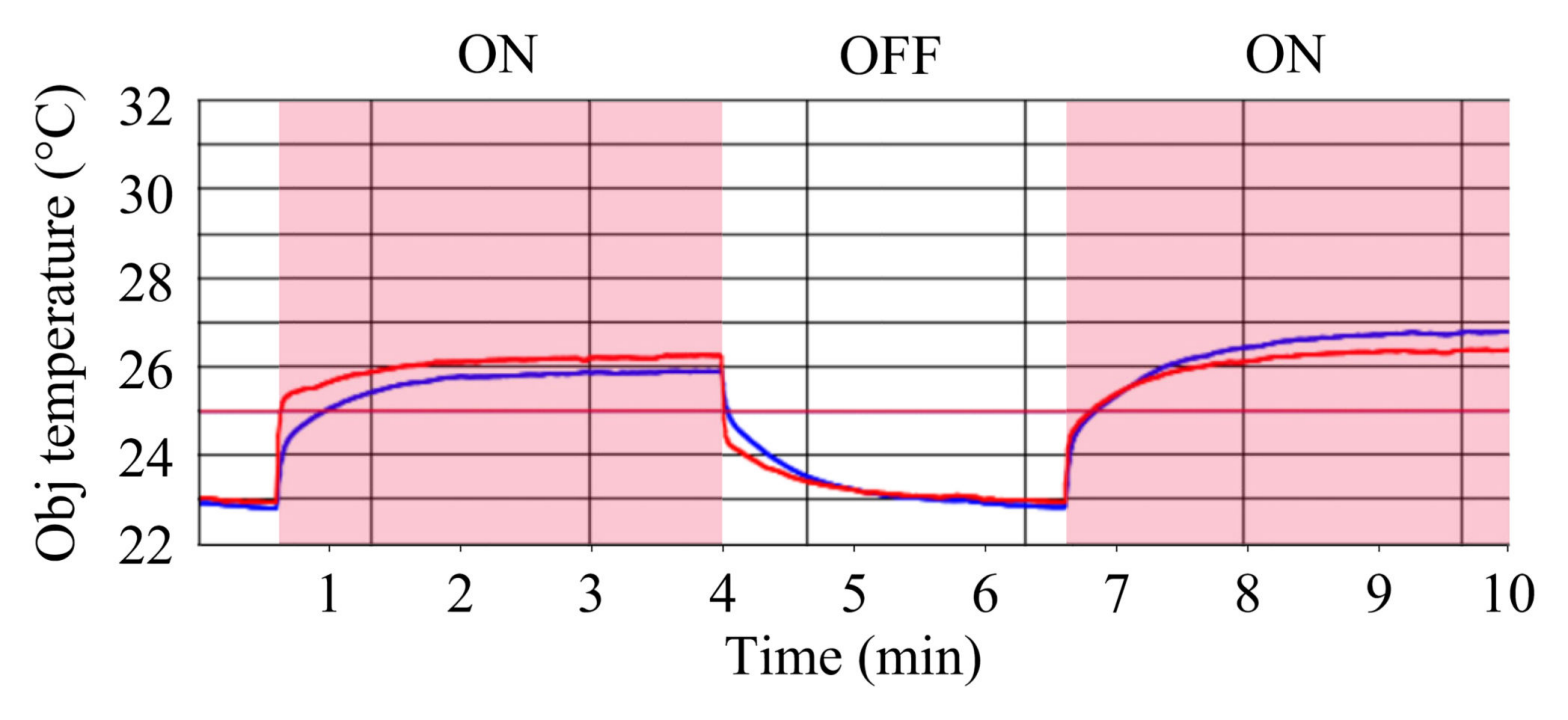
Temperature stabilization for the two piezoelectric elements over the time for two frequencies. The red and blue curve are the temperatures from the two resistance temperature detectors on the piezoelectric elements.

To further investigate the wave mode inside the LDPLUS, the computed slot vibration velocity obtained by the transient simulation is compared to the experimentally obtained velocity of the surface in the slot as measured by LDV, Fig. 7. When the first oscillations are visible in the model after 2.4 μs, the LDV data points are mainly indistinguishable from the noise. At later times, when the longitudinal waves have been focused and directed to the slot, after 4.5 μs, Fig. 6(c), the measured velocities show their first distinct peak (green arrows in Fig. 7). Afterwards, the second largest peak in the measured velocity corresponds to the time in which the transverse waves have built their resonance inside the LDPLUS, which relates to the model peak at 7.4 μs, Fig. 6(d), as shown by the red arrows in Fig. 7. By manually annotating these two peaks in all the measured points along the slot center line, we obtained average times of arrival of 4.6 ± 0.55 μs and ± 7.52 1.42 μs for longitudinal and transverse waves, respectively.

## IV Discussion

The device we presented here aligns well with the current trend in acoustofluidics, aiming to excite strong sound fields to achieve better performance in terms of throughput for various particle sizes, with the overarching goal to target applications in clinical settings. We were able to continuously focus 4.9-μm PS particles at 5 ml/min in a channel of height 150 μm and width 375 μm for a voltage amplitude of 30 V peak to peak and a total input power of 1.5 W. This corresponds to an averaged retention time of 13.5 ms along the actuated area of the channel. Within that time, a particle that starts migrating near the wall travels more than 150 μm, corresponding to an averaged velocity of 11 mm/s. Nevertheless, the focusing does not happen throughout the whole length of the LDPLUS, as clearly shown by Fig. 3, having the two sides which are mostly silent. This means that the particles focus fast only in the acoustic hot spots when flowing though the channel. We cannot easily link the in-flow focusing performance, Fig. 2, with the measured acoustic energy density under the stopped-flow condition, Fig. 3, since in the flow-focusing experiment the system has reached thermal equilibrium, while this is not the case for the stopped-flow experiment. Piezoelectric materials inherently possess dielectric and vibration losses, resulting in temperature rise particularly under intermediate and high power. This inevitably leads to the shift of device resonance frequency. With this in mind, we acquired the images for the in-flow experiments, Fig. 2, only when constant temperature of the piezoelectric elements was reached. However, this was not possible in the stopped-flow experiments to determine *E*_ac_ since the particles settle in the pressure node at a much shorter timescale than the thermal buildup. Thus, the acoustic energy shown in Fig. 3 is somewhat higher than the one in the flow-focusing experiment shown in Fig. 2.

The proposed structure acts as a thermal decoupler between the piezoelectric elements and the microfluidic chip. Aluminum has high heat conductivity, which is roughly 200 times higher than for Pz26 [45]. Hence, heat can be easily dissipated when being conducted from the source (i.e., the piezoelectric elements) towards the chip, especially when air cooling the assembly. In classic bulk acoustic wave devices, the piezoelectric elements are usually glued directly onto the acoustofluidic chip, limiting the ability to dissipate heat before reaching the fluid even when employing air cooling. Having an intermediate material with high thermal conductivity allows higher electrical power to be applied to the piezoelectric elements without the temperature in the chip reaching levels that are harmful to biological samples.

The LDV measurements in the slot can give us an indication of the device vibration at the selected frequency. As seen in the simulation, the longitudinal waves emitted from the piezoelectric elements undergo mode conversion when reaching the big parabola, inevitably inducing transverse waves. It turns out that transverse waves play a significant role in the vibration of the slot, which indicates that the induced wave energy is not fully utilized to purposefully drive the device. Further, the indication of strong mode conversion suggests that the chosen shapes of the two parabolas are likely not optimal. A recent work has explored using an ellipsoidal shape to selectively focus transverse waves [46], but it has not yet been demonstrated for acoustophoresis devices. Nevertheless, the LDPLUS showed higher mean vibration velocity on the slot compared to that of the piezoelectric elements, Fig. 4. However, the velocity magnification was only around 125%, since transverse waves are not focused and directed onto the chip. Even though the LDV measurements in the empty slot can be a clear indication of the device performance at that frequency, we cannot directly link the transducer vibration of the chip-free system to the vibration of the system once the chip has been glued in the slot. The mounted glass-silicon-glass chip and the presence of the coupling layer may change the vibration mode due to the different boundary condition to the device [47].

## V Conclusions

In this work, we showed an alternative approach to excite strong sound fields in acoustofluidic devices. The LDPLUS is a mean to couple the small microfluidic chip with much larger piezoelectric elements, opening the possibility to deliver high power to the device. An averaged acoustic energy density of 1207 J/m^3^ was achieved along the entire actuation zone with a total input power of 1.5 W. This high-energy-density device would enable applications for submicrometric particle focusing if combined with techniques to suppress acoustic streaming [48,49]. In addition, the metallic structure acts not only as a waveguide to focus wave energy, but also as a thermal decoupler between the piezoelectric elements and microfluidic chip, thus preventing temperature rise in the fluid even under relatively high input power. Moreover, we found that transverse waves play a crucial role in delivering wave energy to the chip, though such a design was intended to focus longitudinal waves, which indicates this design has room for optimization. Further study is needed to optimize the transducer design to better focus the wave energy, and to make the whole structure less bulky and thus more suitable for microfluidic applications.

## Supplementary Material

Appendix

## Figures and Tables

**Video 1:**
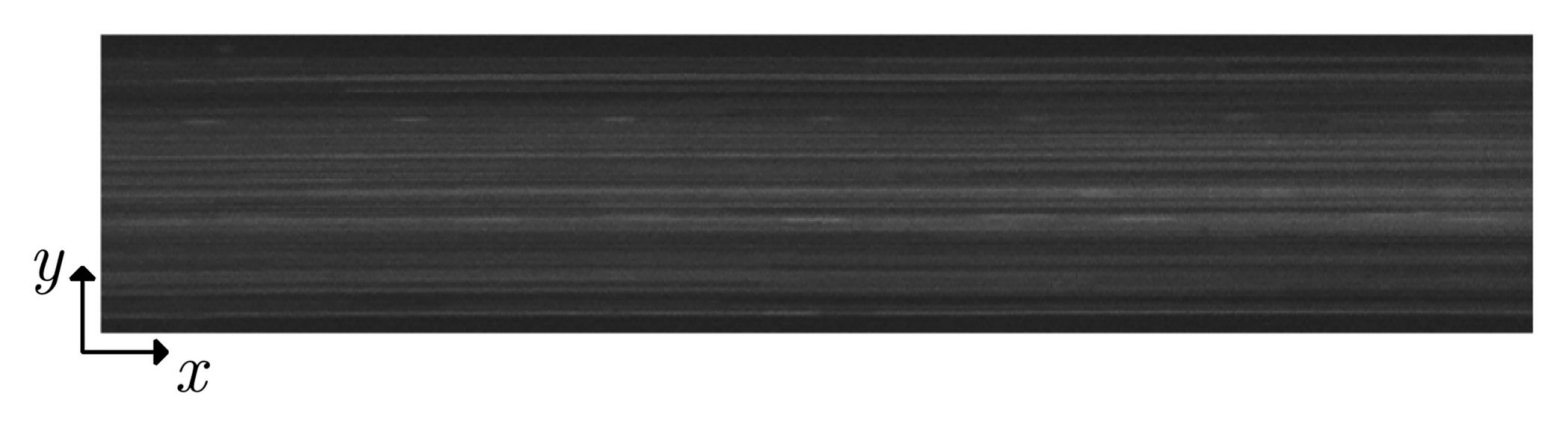
Poor focusing of the fluorescent particles flowing through the microchannel, whose averaged profile is used to calculate the FWHM.

**Video 2:**
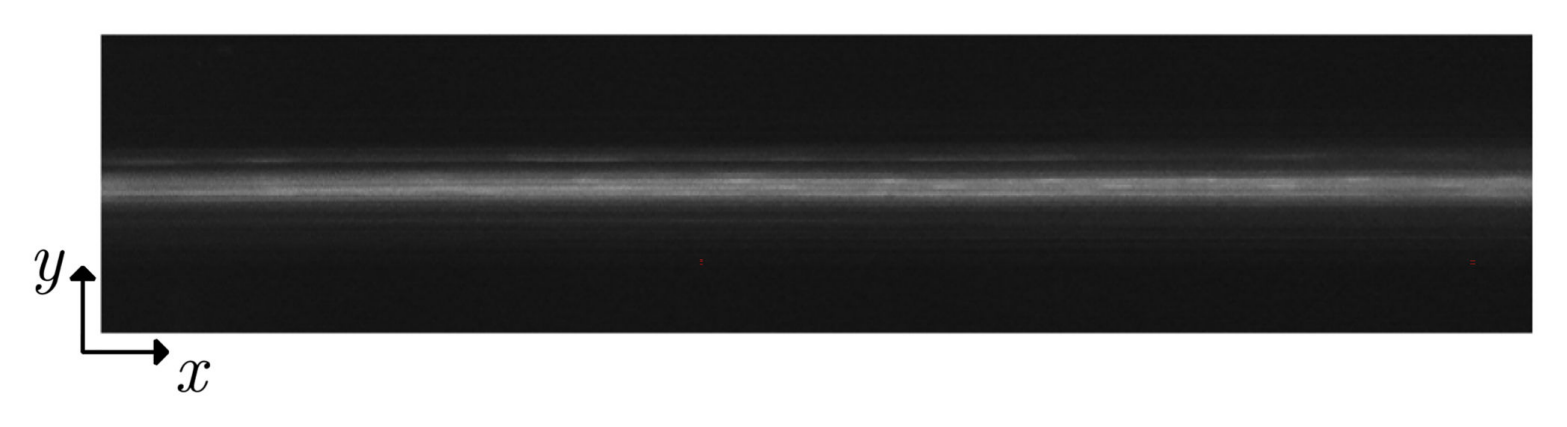
Good focusing of the fluorescent particles flowing through the microchannel, whose averaged profile is used to calculate the FWHM.

**Table I:** Material parameters at room temperature, which are not built into the simulation software. Properties of Pz26 from Ref. [44].

Parameter	Value
*Pz26*	
Mechanical quality factor	3300
Dielectric loss	0.003
*Water*	
Density	997 kg/m^3^
Speed of sound	1481 m/s
Dynamic viscosity	1 mPa s
Bulk viscosity	2.79 mPa s

## Data Availability

The data that support the findings of this article are available upon request.

## References

[1] Petersson K, Jakobsson O, Ohlsson P, Augustsson P, Scheding S, Malm J, Laurell T (2018). Acoustofluidic hematocrit determination. Anal Chim Acta.

[2] Augustsson P, Laurell T (2012). Acoustofluidics 11: Affinity specific extraction and sample decomplexing using continuous flow acoustophoresis. Lab Chip.

[3] Adler SS, Nyong EC, Glabman RA, Choyke PL, Sato N (2022). Cell radiolabeling with acoustophoresis cell washing. Sci Rep.

[4] Augustsson P, Magnusson C, Nordin M, Lilja H, Laurell T (2012). Microfluidic, label-free enrichment of prostate cancer cells in blood based on acoustophoresis. Anal Chem.

[5] Magnusson C, Augustsson P, Undvall Anand E, Lenshof A, Josefsson A, Bjartell A, Ceder Y, Lilja H, Laurell T (2024). Acoustic enrichment of heterogeneous circulating tumor cells and clusters from metastatic prostate cancer patients. Anal Chem.

[6] Li P, Mao Z, Peng Z, Zhou L, Chen Y, Huang PH, Truica I, Drabick JJ, El-Deiry WS, Dao M (2015). Acoustic separation of circulating tumor cells. Proc Natl Acad Sci U S A.

[7] Karthick S, Pradeep PN, Kanchana P, Sen AK (2018). Acoustic impedance-based size-independent isolation of circulating tumour cells from blood using acoustophoresis. Lab Chip.

[8] Augustsson P, Karlsen JT, Su HW, Bruus H, Voldman J (2016). Iso-acoustic focusing of cells for size-insensitive acousto-mechanical phenotyping. Nat Commun.

[9] Urbansky A, Ohlsson P, Lenshof A, Garofalo F, Scheding S, Laurell T (2017). Rapid and effective enrichment of mononuclear cells from blood using acoustophoresis. Sci Rep.

[10] Thévoz P, Adams JD, Shea H, Bruus H, Soh TH (2010). Acoustophoretic synchronization of mammalian cells in microchannels. Anal Chem.

[11] Grenvall C, Augustsson P, Folkenberg JR, Laurell T (2009). Harmonic microchip acoustophoresis: A route to online raw milk sample precondition in protein and lipid content quality control. Anal Chem.

[12] Grenvall C, Folkenberg JR, Augustsson P, Laurell T (2012). Label-free somatic cell cytometry in raw milk using acoustophoresis. Cytometry, Part A.

[13] Ngamsom B, Lopez-Martinez MJ, Raymond J-C, Broyer P, Patel P, Pamme N (2016). On-chip acoustophoretic isolation of microflora including s. Typhimurium from raw chicken, beef and blood samples. J Microbiol Methods.

[14] Rasouli R, Villegas KM, Tabrizian M (2023). Acoustofluidics - changing paradigm in tissue engineering, therapeutics development, and biosensing. Lab Chip.

[15] Burguillos MA, Magnusson C, Nordin M, Lenshof A, Augustsson P, Hansson MJ, Lilja H, Brundin P, Laurell T (2013). Microchannel acoustophoresis does not impact survival or function of microglia, leukocytes or tumor cells. PLoS One.

[16] Hultström J, Manneberg O, Dopf K, Hertz HM, Brismar H, Wiklund M (2007). Proliferation and viability of adherent cells manipulated by standing-wave ultrasound in a microfluidic chip. Ultrasound Med Biol.

[17] Wiklund M (2012). Acoustofluidics 12: Biocompatibility and cell viability in microfluidic acoustic resonators. Lab Chip.

[18] Bruus H (2012). Acoustofluidics 7: The acoustic radiation force on small particles. Lab Chip.

[19] Augustsson P, Barnkob R, Wereley ST, Bruus H, Laurell T (2011). Automated and temperature-controlled micro-PIV measurements enabling long-term-stable microchannel acoustophoresis characterization. Lab Chip.

[20] Adams JD, Ebbesen CL, Barnkob R, Yang AHJ, Soh HT, Bruus H (2012). High-throughput, temperature-controlled microchannel acoustophoresis device made with rapid prototyping. J Micromech Microeng.

[21] Ohlin M, Iranmanesh I, Christakou AE, Wiklund M (2015). Temperature-controlled MPa-pressure ultrasonic cell manipulation in a microfluidic chip. Lab Chip.

[22] Joergensen JH, Bruus H (2021). Theory of pressure acoustics with thermoviscous boundary layers and streaming in elastic cavities. J Acoust Soc Am.

[23] Joergensen JH, Qiu W, Bruus H (2023). Transition from boundary-driven to bulk-driven acoustic streaming due to nonlinear thermoviscous effects at high acoustic energy densities. Phys Rev Lett.

[24] Qiu W, Joergensen JH, Corato E, Bruus H, Augustsson P (2021). Fast microscale acoustic streaming driven by a temperature-gradient-induced nondissipative acoustic body force. Phys Rev Lett.

[25] Bora M, Shusteff M (2015). Efficient coupling of acoustic modes in microfluidic channel devices. Lab Chip.

[26] Qiu W, Baasch T, Laurell T (2022). Enhancement of acoustic energy density in bulk-wave-acoustophoresis devices using side actuation. Phys Rev Appl.

[27] Manneberg O, Svennebring J, Hertz HM, Wiklund M (2008). Wedge transducer design for two-dimensional ultrasonic manipulation in a microfluidic chip. J Micromech Microeng.

[28] Iranmanesh I, Barnkob R, Bruus H, Wiklund M (2013). Tunable-angle wedge transducer for improved acoustophoretic control in a microfluidic chip. J Micromech Microeng.

[29] Mason TJ (2016). Ultrasonic cleaning: An historical perspective. Ultrason Sonochem.

[30] Tsujino J (1995).

[31] Jin M, Murakawa M (2001). Development of a practical ultrasonic vibration cutting tool system. J Mater Process Technol.

[32] Nakamura K (2012). Electronic and optical materials.

[33] Chen K, Irie T, Iijima T, Morita T (2019). Double-parabolic-reflectors acoustic waveguides for high-power medical ultrasound. Sci Rep.

[34] Chen K, Irie T, Iijima T, Morita T (2020). Wideband multimode excitation by a double-parabolic-reflector ultrasonic transducer. IEEE Trans Ultrason Ferroelectr Freq Control.

[35] Gor’kov LP (1962). On the forces acting on a small particle in an acoustical field in an ideal fluid. Sov Phys Dokl.

[36] Barnkob R, Augustsson P, Laurell T, Bruus H (2012). Acoustic radiation- and streaming-induced microparticle velocities determined by microparticle image velocimetry in an ultrasound symmetry plane. Phys Rev E.

[37] Rayleigh L (1884). On the circulation of air observed in Kundt’s tubes, and on some allied acoustical problems. Philos Trans R Soc London.

[38] Barnkob R, Augustsson P, Laurell T, Bruus H (2010). Measuring the local pressure amplitude in microchannel acoustophoresis. Lab Chip.

[39] Faxén H (1922). Der widerstand gegen die bewegung einer starren kugel in einer zähen flüssigkeit, die zwischen zwei parallelen ebenen wänden eingeschlossen ist. Ann Phys.

[40] https://defocustracking.com/ https://defocustracking.com/.

[41] Barnkob R, Rossi M (2020). General defocusing particle tracking: Fundamentals and uncertainty assessment. Exp Fluids.

[42] Rossi M, Barnkob R (2020). A fast and robust algorithm for general defocusing particle tracking. Meas Sci Technol.

[43] Comsol multiphysics 6.1.

[44] Ferroperm piezoceramics.

[45] Physik instrumente (pi).

[46] Ieiri S, Yamada K, Sasamura T, Itoh S, Kasashima T, Morita T (2024). An ultrasonic transducer focusing ultrasound into a thin waveguide by two elliptical reflectors. Appl Phys Express.

[47] Bodé WN, Bruus H (2021). Numerical study of the coupling layer between transducer and chip in acoustofluidic devices. J Acoust Soc Am.

[48] Bach JS, Bruus H (2020). Suppression of acoustic streaming in shape-optimized channels. Phys Rev Lett.

[49] Karlsen JT, Qiu W, Augustsson P, Bruus H (2018). Acoustic streaming and its suppression in inhomogeneous fluids. Phys Rev Lett.

