# Supplementary material for: High-energy-density acoustofluidic device using a double-parabolic ultrasonic transducer
Source: Phys Rev Appl. Author manuscript; Available in PMC 2025 Sep 24. (PMC7618166; doi:10.1103/physrevapplied.23.024031)
Supplement: Appendix [file EMS208793-supplement-Appendix.pdf]

APPENDIX A

Figure 8 shows the temperature stabilization of the two piezoelectric elements for two different frequencies. The image acquisition started once the device reached a steady thermal state, namely a temperature variation of less than 0.2 °C on both piezoelectric elements. Once the images had been acquired, we turned the sound off and let the device cool to room temperature. Then the process started over with another frequency.
